# 4-[5-(4-Pyrid­yl)-1,3,4-oxadiazol-2-yl]pyridine *N*-oxide–isophthalic acid (1/1)

**DOI:** 10.1107/S1600536808012622

**Published:** 2008-05-07

**Authors:** Gui-Ge Hou, Li-Li Liu, Jian-Ping Ma, Ru-Qi Huang, Yu-Bin Dong

**Affiliations:** a College of Chemistry, Chemical Engineering and Materials Science, Shandong Normal University, Jinan 250014, People’s Republic of China

## Abstract

The title compound, C_12_H_8_N_4_O_2_·C_8_H_6_O_4_, was synthesized from 4-[5-(4-pyrid­yl)-1,3,4-oxadiazol-2-yl]pyridine *N*-oxide and isophthalic acid. The two mol­ecules are linked through O—H⋯O and O—H⋯N hydrogen bonds. Weak intra­molecular π–π inter­actions between the two hydrogen-bonded chains result in the formation a one-dimensional supra­molecular curved tape (the face-to-face distance between the pyridine *N*-oxide ring and the benzene ring is 3.7 Å).

## Related literature

For related literature, see: Beckmann & Jänicke (2006 ▶); Dong *et al.* (2003 ▶); Du *et al.* (2006 ▶); Hunter (1994 ▶); Kitagawa *et al.* (2004 ▶); Long *et al.* (2004 ▶); Lu *et al.* (1997 ▶); Ma *et al.* (2005 ▶); Ren *et al.* (1995 ▶); Tan *et al.* (2006 ▶).
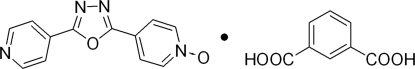


## Experimental

### 

#### Crystal data

C_12_H_8_N_4_O_2_·C_8_H_6_O_4_
*M* *_r_* = 406.35
Triclinic, 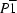

*a* = 7.0993 (18) Å
*b* = 7.1770 (19) Å
*c* = 19.823 (5) Å
α = 93.003 (4)°
β = 98.481 (3)°
γ = 112.745 (4)°
*V* = 914.6 (4) Å^3^
*Z* = 2
Mo *K*α radiation
μ = 0.11 mm^−1^
*T* = 298 (2) K
0.30 × 0.15 × 0.06 mm

#### Data collection

Bruker SMART APEX diffractometer
Absorption correction: none
4661 measured reflections
3189 independent reflections
1972 reflections with *I* > 2σ(*I*)
*R*_int_ = 0.027

#### Refinement

*R*[*F* ^2^ > 2σ(*F* ^2^)] = 0.052
*wR*(*F* ^2^) = 0.132
*S* = 0.98
3189 reflections
272 parameters
H-atom parameters constrained
Δρ_max_ = 0.30 e Å^−3^
Δρ_min_ = −0.15 e Å^−3^

### 

Data collection: *SMART* (Bruker 2000 ▶); cell refinement: *SMART*; data reduction: *SAINT* (Bruker 2000 ▶); program(s) used to solve structure: *SHELXS97* (Sheldrick, 2008 ▶); program(s) used to refine structure: *SHELXL97* (Sheldrick, 2008 ▶); molecular graphics: *SHELXTL* (Sheldrick, 2008 ▶); software used to prepare material for publication: *SHELXTL*.

## Supplementary Material

Crystal structure: contains datablocks I, global. DOI: 10.1107/S1600536808012622/bv2095sup1.cif

Structure factors: contains datablocks I. DOI: 10.1107/S1600536808012622/bv2095Isup2.hkl

Additional supplementary materials:  crystallographic information; 3D view; checkCIF report

## Figures and Tables

**Table 1:** Hydrogen-bond geometry (Å, °)

*D*—H⋯*A*	*D*—H	H⋯*A*	*D*⋯*A*	*D*—H⋯*A*
O1—H1⋯N4	0.82	1.82	2.640 (2)	171
O3—H3⋯O5^i^	0.82	1.72	2.521 (3)	163

Symmetry code: (i) 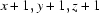.

**Fig. 1.**
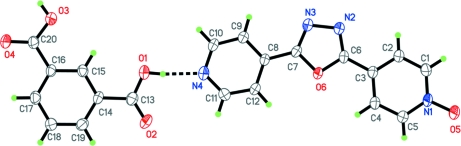
: The structure of (1), with 30% probability displacement ellipsoids (arbitrary spheres for H atoms).

**Fig. 2.**
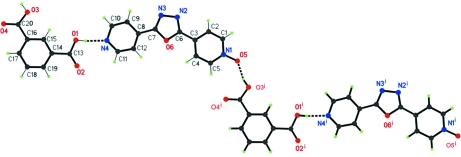
: A view of the hydrogen-bonded chains observed in the crystal structure of (1).

**Fig. 3.**
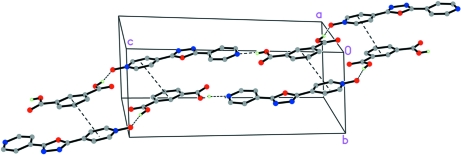
: The crystal packing of (1) via weak π-π interactions, and the face-to-face distance is 3.7 Å.

**Fig. 4.**
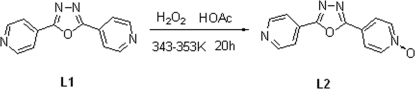
The formation of L2.

## supplementary crystallographic information

### Comment

Rigid pyridyl compounds have been considered as good hydrogen bond acceptors. These compounds include 4,4-bipyridine (Lu *et al.*, 1997; Kitagawa *et al.*, 2004; Tan *et al.*, 2006), 1,2-bis(4-pyridyl)ethyne (Beckmann & Jänicke, 2006), 4,4'-bipyridine-*N*,*N*'-dioxide (Long *et al.*, 2004; Ma *et al.*, 2005), and other similar molecules. We recently prepared the rigid bent compound, 2-(4-pyridyl)-5-(4-pyridyl *N*-oxide)-1,3,4-oxadiazole, (L2). It has a rigid 144° angle because of the bridging five-membered oxadiazole ring. In addition, isophthalic acid is a good hydrogen-bonding donor and its two carboxylic acid moieties make an angle of 120°. Supramolecular systems with novel architecture may result because of their specific geometry. Accordingly, when L2 reacted with isophthalic acid in a mixed CH_2_Cl_2_/CH_3_OH solution, yellow crystals formed and its structure is reported here.

The molecular structure of (1) is shown in Fig. 1. The asymmetric unit contains one L2 molecule and one isophthalic acid molecule. O···H—O and N···H—O hydrogen bonds (Fig. 3) are formed by the –COOH groups of the isophthalic acid and the pyridyl-N-oxide O atom and N_pyridine_ atom, (Table 1). The dihedral angles between the benzene ring of isophthalic acid and pyridyl-N-oxide ring and pyridyl ring are 1.4° and 7.0°, respectively, which are similar to previously reported experimental values (Du *et al.*, 2006; Tan *et al.*, 2006). In addition to the specific geometry of the oxadiazole-containing rigid curved organic ligand L2, the molecules in (1) connect with each other generating one-dimensional extended zigzag chains, which have not been obtained using normal linear rigid bidentate organic ligands (Dong & Ma, 2003).

In the solid state, the crystal packing view of (1) shows a pair of zigzag chains which stack *via* aromatic π-π interactions (Fig. 3). The face-to-face distance between the pyridine-N-oxide ring and benzene ring from isophthalic acid is 3.7 Å. The shortest close contact is 3.447 (6) Å. Although these values are typical for aromatic π-π stacking interactions, compared with the strong π-π interactions (Hunter, 1994) they are comparatively weak. Two adjacent chains are further linked *via* intramolecular π-π stacking interactions to construct a one-dimensional supramolecular curved tape (Fig. 3). These weak intramolecular π-π interactions and crucial hydrogen bonds enhance the stability of the compound (1).

### Experimental

L1 (2,5-bis(4-pyridyl)-1,3,4-oxadiazole) was prepared according to literature methods (Ren *et al.*, 1995). L1 (1.1 g, 5 mmol), 1.0 ml hydrogen dioxide 30% solution and 3.0 ml acetic acid were mixed and refluxed at 343–353 K for 20 h. After removal of solvent under vacuum, the residue was purified on a silica gel column using CH_2_Cl_2_/CH_3_OH (15:1, *v*/*v*) as the eluent to afford L2, Yield: 38%. Mp: 501–503 K. 1H NMR (DMSO, 300 MHz, p.p.m.): δ 8.89–8.91 (d, 2H, 2C_5_H_4_N), 8.34–8.37 (d, 2H, 2C_5_H_4_NO), 7.99–8.06 (m, 4H, 2C_5_H_4_N, 2C_5_H_4_NO). IR (KBr pellet cm^-1^): 3423(*s*), 1620(*m*), 1563(*m*), 1537(*m*), 1475(*s*), 1439(*s*), 1407(*s*), 1272(*s*), 1174(*s*), 1111(*m*), 965(*m*), 848(*m*), 740(*m*), 703(*s*), 644(*s*), 509(*m*).

A CH_2_Cl_2_ and CH_3_OH solution (10 ml, 1:1, *v*/*v*) of L2 (24 mg, 0.1 mmol) and isophthalic acid (16.6 mg, 0.1 mmol) kept at room temperature. After a few days yellow block crystals (1) were obtained (36 mg). Yield: 89%.

### Refinement

H atoms on O atoms were located in a difference Fourier map and refined as riding in their as-found relative positions, with *U*_iso_(H) = 1.5*U*_eq_(O). Other H atoms were placed in calculated positions, with C—H = 0.93 Å, and refined in riding mode, with *U*_iso_(H) = 1.2*U*_eq_(C) (aromatic).

### Crystal data

**Table:**

C_12_H_8_N_4_O_2_·C_8_H_6_O_4_	*Z* = 2
*M**_r_* = 406.35	*F*_000_ = 420
Triclinic, *P*1	*D*_x_ = 1.475 Mg m^−^^3^
Hall symbol: -P 1	Mo *K*α radiation λ = 0.71073 Å
*a* = 7.0993 (18) Å	Cell parameters from 893 reflections
*b* = 7.1770 (19) Å	θ = 3.2–21.1º
*c* = 19.823 (5) Å	µ = 0.11 mm^−^^1^
α = 93.003 (4)º	*T* = 298 (2) K
β = 98.481 (3)º	Bar, colourless
γ = 112.745 (4)º	0.30 × 0.15 × 0.06 mm
*V* = 914.6 (4) Å^3^	

### Data collection

**Table:**

Bruker SMART APEX diffractometer	1972 reflections with *I* > 2σ(*I*)
Radiation source: fine-focus sealed tube	*R*_int_ = 0.027
Monochromator: graphite	θ_max_ = 25.0º
*T* = 298(2) K	θ_min_ = 3.1º
φ and ω scans	*h* = −8→8
Absorption correction: none	*k* = −8→8
4661 measured reflections	*l* = −17→23
3189 independent reflections	

### Refinement

**Table:**

Refinement on *F*^2^	Secondary atom site location: difference Fourier map
Least-squares matrix: full	Hydrogen site location: inferred from neighbouring sites
*R*[*F*^2^ > 2σ(*F*^2^)] = 0.052	H-atom parameters constrained
*wR*(*F*^2^) = 0.132	*w* = 1/[σ^2^(*F*_o_^2^) + (0.0637*P*)^2^] where *P* = (*F*_o_^2^ + 2*F*_c_^2^)/3
*S* = 0.98	(Δ/σ)_max_ = 0.001
3189 reflections	Δρ_max_ = 0.30 e Å^−^^3^
272 parameters	Δρ_min_ = −0.15 e Å^−^^3^
Primary atom site location: structure-invariant direct methods	Extinction correction: none

### Special details

**Table:**

Geometry. All e.s.d.'s (except the e.s.d. in the dihedral angle between two l.s. planes) are estimated using the full covariance matrix. The cell e.s.d.'s are taken into account individually in the estimation of e.s.d.'s in distances, angles and torsion angles; correlations between e.s.d.'s in cell parameters are only used when they are defined by crystal symmetry. An approximate (isotropic) treatment of cell e.s.d.'s is used for estimating e.s.d.'s involving l.s. planes.
Refinement. Refinement of *F*^2^ against ALL reflections. The weighted *R*-factor *wR* and goodness of fit *S* are based on *F*^2^, conventional *R*-factors *R* are based on *F*, with *F* set to zero for negative *F*^2^. The threshold expression of *F*^2^ > σ(*F*^2^) is used only for calculating *R*-factors(gt) *etc*. and is not relevant to the choice of reflections for refinement. *R*-factors based on *F*^2^ are statistically about twice as large as those based on *F*, and *R*- factors based on ALL data will be even larger.

### Fractional atomic coordinates and isotropic or equivalent isotropic displacement parameters (Å^2^)

**Table:**

	*x*	*y*	*z*	*U*_iso_*/*U*_eq_	
O1	0.7708 (3)	0.9021 (3)	0.64281 (9)	0.0693 (6)	
O2	1.0221 (3)	0.8709 (4)	0.59557 (10)	0.0861 (7)	
O5	−0.2941 (3)	0.2221 (4)	−0.02763 (9)	0.0882 (8)	
O3	0.8643 (3)	1.0843 (3)	0.88695 (9)	0.0738 (6)	
H3	0.8309	1.1276	0.9203	0.111*	
O4	1.1774 (3)	1.2181 (3)	0.95238 (9)	0.0726 (6)	
C1	−0.4071 (4)	0.2792 (4)	0.06980 (13)	0.0592 (8)	
H1A	−0.5408	0.2374	0.0446	0.071*	
C2	−0.3697 (4)	0.3405 (4)	0.13821 (12)	0.0528 (7)	
H2	−0.4781	0.3399	0.1595	0.063*	
C3	−0.1721 (4)	0.4035 (4)	0.17649 (11)	0.0427 (6)	
C4	−0.0155 (4)	0.4016 (4)	0.14322 (12)	0.0515 (7)	
H4	0.1189	0.4429	0.1678	0.062*	
C5	−0.0572 (4)	0.3385 (4)	0.07365 (12)	0.0558 (7)	
H5A	0.0489	0.3375	0.0513	0.067*	
C6	−0.1345 (4)	0.4706 (4)	0.24973 (12)	0.0436 (6)	
C7	0.0337 (4)	0.5784 (4)	0.35129 (11)	0.0431 (6)	
C8	0.2083 (4)	0.6463 (4)	0.40831 (11)	0.0415 (6)	
C9	0.1803 (4)	0.7064 (4)	0.47216 (12)	0.0496 (7)	
H9	0.0533	0.7076	0.4782	0.059*	
C10	0.3421 (4)	0.7639 (4)	0.52633 (12)	0.0520 (7)	
H10	0.3205	0.8000	0.5694	0.062*	
C11	0.5555 (4)	0.7154 (4)	0.45907 (13)	0.0551 (7)	
H11	0.6856	0.7205	0.4542	0.066*	
C12	0.4001 (4)	0.6505 (4)	0.40169 (12)	0.0490 (7)	
H12	0.4245	0.6105	0.3596	0.059*	
C13	0.9602 (4)	0.9161 (4)	0.64414 (14)	0.0521 (7)	
C14	1.0945 (4)	0.9948 (4)	0.71368 (11)	0.0453 (6)	
C15	1.0149 (4)	1.0271 (4)	0.77116 (12)	0.0451 (6)	
H15	0.8738	0.9975	0.7670	0.054*	
C16	1.1455 (4)	1.1037 (4)	0.83507 (11)	0.0436 (6)	
C17	1.3553 (4)	1.1470 (4)	0.84013 (13)	0.0533 (7)	
H17	1.4443	1.1995	0.8822	0.064*	
C18	1.4324 (4)	1.1130 (4)	0.78334 (13)	0.0577 (8)	
H18	1.5734	1.1419	0.7874	0.069*	
C19	1.3043 (4)	1.0369 (4)	0.72065 (13)	0.0542 (7)	
H19	1.3586	1.0134	0.6827	0.065*	
C20	1.0666 (4)	1.1418 (4)	0.89757 (13)	0.0509 (7)	
N1	−0.2515 (3)	0.2788 (3)	0.03830 (10)	0.0569 (6)	
N2	−0.2670 (3)	0.4826 (3)	0.28576 (10)	0.0511 (6)	
N3	−0.1552 (3)	0.5550 (3)	0.35274 (10)	0.0529 (6)	
N4	0.5295 (3)	0.7709 (3)	0.52071 (10)	0.0525 (6)	
H1	0.7016	0.8526	0.6043	0.079*	
O6	0.0597 (2)	0.5275 (2)	0.28698 (7)	0.0451 (5)	

### Atomic displacement parameters (Å^2^)

**Table:**

	*U*^11^	*U*^22^	*U*^33^	*U*^12^	*U*^13^	*U*^23^
O1	0.0608 (14)	0.0982 (17)	0.0411 (11)	0.0313 (12)	−0.0073 (9)	−0.0095 (10)
O2	0.0854 (16)	0.128 (2)	0.0449 (12)	0.0492 (15)	0.0062 (11)	−0.0239 (12)
O5	0.0759 (15)	0.157 (2)	0.0339 (12)	0.0592 (15)	−0.0082 (10)	−0.0223 (12)
O3	0.0545 (13)	0.1118 (18)	0.0450 (12)	0.0277 (12)	0.0025 (9)	−0.0144 (11)
O4	0.0630 (13)	0.1144 (18)	0.0341 (11)	0.0374 (12)	−0.0070 (9)	−0.0135 (11)
C1	0.0494 (17)	0.077 (2)	0.0459 (17)	0.0259 (15)	−0.0048 (13)	−0.0079 (14)
C2	0.0514 (17)	0.0658 (19)	0.0379 (16)	0.0239 (14)	0.0000 (12)	−0.0024 (13)
C3	0.0490 (16)	0.0424 (16)	0.0352 (14)	0.0190 (12)	0.0020 (12)	0.0006 (11)
C4	0.0498 (17)	0.0596 (19)	0.0377 (16)	0.0182 (14)	−0.0013 (12)	−0.0006 (12)
C5	0.0523 (18)	0.075 (2)	0.0405 (16)	0.0282 (15)	0.0051 (13)	0.0002 (14)
C6	0.0501 (17)	0.0421 (16)	0.0331 (14)	0.0167 (13)	−0.0027 (12)	0.0002 (11)
C7	0.0534 (17)	0.0443 (16)	0.0326 (15)	0.0210 (13)	0.0079 (12)	0.0010 (11)
C8	0.0474 (16)	0.0436 (16)	0.0313 (14)	0.0182 (12)	0.0019 (11)	0.0009 (11)
C9	0.0487 (16)	0.0590 (18)	0.0375 (15)	0.0193 (14)	0.0060 (12)	−0.0018 (12)
C10	0.0591 (19)	0.0614 (19)	0.0319 (15)	0.0225 (15)	0.0052 (13)	−0.0025 (12)
C11	0.0491 (17)	0.066 (2)	0.0508 (18)	0.0258 (15)	0.0033 (14)	0.0032 (14)
C12	0.0551 (18)	0.0575 (18)	0.0338 (15)	0.0228 (14)	0.0073 (13)	−0.0006 (12)
C13	0.0625 (19)	0.0471 (18)	0.0468 (18)	0.0236 (14)	0.0068 (14)	0.0024 (13)
C14	0.0574 (17)	0.0467 (17)	0.0315 (14)	0.0233 (13)	0.0020 (12)	−0.0004 (11)
C15	0.0472 (15)	0.0468 (16)	0.0417 (15)	0.0221 (13)	0.0003 (12)	0.0039 (12)
C16	0.0492 (16)	0.0482 (16)	0.0329 (14)	0.0220 (13)	−0.0005 (12)	0.0029 (11)
C17	0.0537 (18)	0.0604 (19)	0.0428 (16)	0.0251 (15)	−0.0042 (13)	−0.0001 (13)
C18	0.0508 (17)	0.073 (2)	0.0485 (18)	0.0270 (16)	0.0037 (14)	0.0004 (15)
C19	0.0609 (19)	0.0605 (19)	0.0458 (17)	0.0285 (15)	0.0126 (14)	0.0029 (13)
C20	0.0509 (18)	0.0600 (19)	0.0408 (16)	0.0251 (14)	−0.0007 (13)	0.0014 (13)
N1	0.0585 (16)	0.0779 (18)	0.0301 (13)	0.0295 (13)	−0.0054 (11)	−0.0088 (11)
N2	0.0505 (14)	0.0635 (16)	0.0366 (13)	0.0230 (12)	0.0021 (10)	−0.0024 (10)
N3	0.0525 (15)	0.0684 (16)	0.0351 (13)	0.0244 (12)	0.0016 (10)	−0.0013 (10)
N4	0.0582 (15)	0.0540 (15)	0.0382 (13)	0.0189 (12)	−0.0012 (11)	0.0011 (10)
O6	0.0486 (11)	0.0548 (11)	0.0293 (9)	0.0211 (9)	0.0009 (8)	−0.0035 (7)

### Geometric parameters (Å, °)

**Table:**

O1—C13	1.306 (3)	C8—C12	1.377 (3)
O1—H1	0.8221	C8—C9	1.382 (3)
O2—C13	1.195 (3)	C9—C10	1.366 (3)
O5—N1	1.303 (2)	C9—H9	0.9300
O3—C20	1.312 (3)	C10—N4	1.333 (3)
O3—H3	0.8200	C10—H10	0.9300
O4—C20	1.205 (3)	C11—N4	1.326 (3)
C1—N1	1.347 (3)	C11—C12	1.380 (3)
C1—C2	1.358 (3)	C11—H11	0.9300
C1—H1A	0.9300	C12—H12	0.9300
C2—C3	1.381 (3)	C13—C14	1.497 (3)
C2—H2	0.9300	C14—C19	1.384 (3)
C3—C4	1.378 (3)	C14—C15	1.391 (3)
C3—C6	1.457 (3)	C15—C16	1.398 (3)
C4—C5	1.380 (3)	C15—H15	0.9300
C4—H4	0.9300	C16—C17	1.385 (3)
C5—N1	1.343 (3)	C16—C20	1.487 (3)
C5—H5A	0.9300	C17—C18	1.372 (3)
C6—N2	1.287 (3)	C17—H17	0.9300
C6—O6	1.358 (3)	C18—C19	1.372 (3)
C7—N3	1.290 (3)	C18—H18	0.9300
C7—O6	1.366 (3)	C19—H19	0.9300
C7—C8	1.454 (3)	N2—N3	1.400 (3)
			
C13—O1—H1	109.3	C8—C12—C11	118.4 (2)
C20—O3—H3	109.5	C8—C12—H12	120.8
N1—C1—C2	120.3 (2)	C11—C12—H12	120.8
N1—C1—H1A	119.8	O2—C13—O1	124.3 (3)
C2—C1—H1A	119.8	O2—C13—C14	122.8 (3)
C1—C2—C3	120.7 (2)	O1—C13—C14	112.8 (2)
C1—C2—H2	119.7	C19—C14—C15	119.2 (2)
C3—C2—H2	119.7	C19—C14—C13	118.7 (2)
C4—C3—C2	118.0 (2)	C15—C14—C13	122.0 (2)
C4—C3—C6	122.1 (2)	C14—C15—C16	120.4 (2)
C2—C3—C6	119.9 (2)	C14—C15—H15	119.8
C3—C4—C5	120.3 (2)	C16—C15—H15	119.8
C3—C4—H4	119.8	C17—C16—C15	118.9 (2)
C5—C4—H4	119.8	C17—C16—C20	119.1 (2)
N1—C5—C4	119.7 (2)	C15—C16—C20	122.0 (2)
N1—C5—H5A	120.1	C18—C17—C16	120.3 (2)
C4—C5—H5A	120.1	C18—C17—H17	119.8
N2—C6—O6	113.3 (2)	C16—C17—H17	119.8
N2—C6—C3	127.5 (2)	C19—C18—C17	120.8 (3)
O6—C6—C3	119.1 (2)	C19—C18—H18	119.6
N3—C7—O6	112.1 (2)	C17—C18—H18	119.6
N3—C7—C8	127.9 (2)	C18—C19—C14	120.2 (2)
O6—C7—C8	120.0 (2)	C18—C19—H19	119.9
C12—C8—C9	118.4 (2)	C14—C19—H19	119.9
C12—C8—C7	122.7 (2)	O4—C20—O3	123.4 (2)
C9—C8—C7	118.8 (2)	O4—C20—C16	123.4 (2)
C10—C9—C8	119.0 (2)	O3—C20—C16	113.2 (2)
C10—C9—H9	120.5	O5—N1—C5	120.9 (2)
C8—C9—H9	120.5	O5—N1—C1	118.2 (2)
N4—C10—C9	123.4 (2)	C5—N1—C1	120.9 (2)
N4—C10—H10	118.3	C6—N2—N3	105.6 (2)
C9—C10—H10	118.3	C7—N3—N2	106.81 (19)
N4—C11—C12	123.7 (2)	C11—N4—C10	117.1 (2)
N4—C11—H11	118.2	C6—O6—C7	102.14 (18)
C12—C11—H11	118.2		
			
N1—C1—C2—C3	0.2 (4)	C14—C15—C16—C20	179.5 (2)
C1—C2—C3—C4	−0.2 (4)	C15—C16—C17—C18	−0.7 (4)
C1—C2—C3—C6	179.4 (2)	C20—C16—C17—C18	179.9 (2)
C2—C3—C4—C5	0.1 (4)	C16—C17—C18—C19	0.4 (4)
C6—C3—C4—C5	−179.5 (2)	C17—C18—C19—C14	0.6 (4)
C3—C4—C5—N1	0.0 (4)	C15—C14—C19—C18	−1.2 (4)
C4—C3—C6—N2	178.5 (3)	C13—C14—C19—C18	178.7 (2)
C2—C3—C6—N2	−1.1 (4)	C17—C16—C20—O4	3.7 (4)
C4—C3—C6—O6	−2.0 (4)	C15—C16—C20—O4	−175.7 (3)
C2—C3—C6—O6	178.4 (2)	C17—C16—C20—O3	−176.5 (2)
N3—C7—C8—C12	174.8 (2)	C15—C16—C20—O3	4.1 (4)
O6—C7—C8—C12	−4.2 (4)	C4—C5—N1—O5	178.7 (2)
N3—C7—C8—C9	−4.2 (4)	C4—C5—N1—C1	0.0 (4)
O6—C7—C8—C9	176.9 (2)	C2—C1—N1—O5	−178.8 (2)
C12—C8—C9—C10	−1.1 (4)	C2—C1—N1—C5	0.0 (4)
C7—C8—C9—C10	177.9 (2)	O6—C6—N2—N3	0.5 (3)
C8—C9—C10—N4	2.0 (4)	C3—C6—N2—N3	−179.9 (2)
C9—C8—C12—C11	−0.3 (4)	O6—C7—N3—N2	0.4 (3)
C7—C8—C12—C11	−179.3 (2)	C8—C7—N3—N2	−178.6 (2)
N4—C11—C12—C8	1.2 (4)	C6—N2—N3—C7	−0.5 (3)
O2—C13—C14—C19	6.8 (4)	C12—C11—N4—C10	−0.5 (4)
O1—C13—C14—C19	−173.4 (2)	C9—C10—N4—C11	−1.1 (4)
O2—C13—C14—C15	−173.3 (3)	N2—C6—O6—C7	−0.3 (3)
O1—C13—C14—C15	6.5 (4)	C3—C6—O6—C7	−179.9 (2)
C19—C14—C15—C16	0.9 (4)	N3—C7—O6—C6	0.0 (3)
C13—C14—C15—C16	−179.0 (2)	C8—C7—O6—C6	179.0 (2)
C14—C15—C16—C17	0.1 (4)		

### Hydrogen-bond geometry (Å, °)

**Table:**

*D*—H···*A*	*D*—H	H···*A*	*D*···*A*	*D*—H···*A*
O1—H1···N4	0.82	1.82	2.640 (2)	171
O3—H3···O5^i^	0.82	1.72	2.521 (3)	163

Symmetry codes: (i) *x*+1, *y*+1, *z*+1.
